# A Mechanistic Understanding of a Binary Additive System to Synergistically Boost Efficiency in All-Polymer Solar Cells

**DOI:** 10.1038/srep18024

**Published:** 2015-12-11

**Authors:** Yu Jin Kim, Sunyong Ahn, Dong Hwan Wang, Chan Eon Park

**Affiliations:** 1 POSTECH Organic Electronics Laboratory, Department of Chemical Engineering, Pohang University of Science and Technology, Pohang, 790-784, Republic of Korea; 2 School of Integrative Engineering, Chung-Ang University, 84 Heukseok-Ro, Dongjak-gu, Seoul, 156-756, Republic of Korea

## Abstract

All-polymer solar cells are herein presented utilizing the PBDTTT-CT donor and the P(NDI2OD-T2) acceptor with 1,8-diiodooctane (DIO) and 1-chloronaphthalene (CN) binary solvent additives. A systematic study of the polymer/polymer bulk heterojunction photovoltaic cells processed from the binary additives revealed that the microstructures and photophysics were quite different from those of a pristine system. The combination of DIO and CN with a DIO/CN ratio of 3:1 (3 vol% DIO, 1 vol% CN and 96 vol% *o*-DCB) led to suitable penetrating polymer networks, efficient charge generation and balanced charge transport, which were all beneficial to improving the efficiency. This improvement is attributed to increase in power conversion efficiency from 2.81% for a device without additives to 4.39% for a device with the binary processing additives. A detailed investigation indicates that the changes in the polymer:polymer interactions resulted in the formation of a percolating nasnoscale morphology upon processing with the binary additives. Depth profile measurements with a two-dimensional grazing incidence wide-angle X-ray scattering confirm this optimum phase feature. Furthermore impedance spectroscopy also finds evidence for synergistically boosting the device performance.

Solution processed thin-film solar cells have the potential to deliver cheap, clean energy by converting incident solar flux into an electrical current. The thin-film solar cells that have been most extensively studied have a bulk heterojunction (BHJ) active layer in which an electron donor polymer is mixed with an acceptor with a low molecular weight^1^,^2^,^3^. To date, the most successful electron acceptors used in these devices are based on fullerene derivatives, including [6,6]-phenyl-C71-butyric acid methyl ester (PC_71_BM) in particular. However, fullerene derivatives are expensive to produce and have relatively low absorption coefficients within the terrestrial solar spectrum^4^,^5^. Therefore, there is significant interest to develop alternative non-fullerene-based electron acceptors^6^,^7^,^8^.

To date, several classes of solution-processable non-fullerene acceptors have been considered. For example, recent reports have studied photovoltaics devices based on a poly{[N,N′-bis(2-octyldodecyl)-naphthalene-1,4,5,8-bis(dicarboximide)-2,6-diyl]-alt-5,5′-(2,2′-bithiophene)} [P(NDI2OD-T2)] acceptor. In particular, Facchetti, Ito, and Marks have shown that all-polymer solar cells using the P(NDI2OD-T2) compound are able to harvest a large portion of sun light with high absorption coefficients in the visible and near-infrared wavelengths^9^,^10^,^11^.

However, the PCEs of these all-polymer solar cells are still inferior to those of polymer:fullerene solar cells, and only a few papers have reported PCEs near 6–7%. The main restriction to obtain high-efficiency all-polymer solar cells is the poor phase separation caused by entanglement of the polymer chains in the all-polymer blend films. In the BHJ composite, the phase-separated domains provide not only the interfaces for charge separation of the photogenerated excitons, but also serve as percolation pathways for the separated charge carriers that are to be transported to the respective electrodes^12^,^13^,^14^. Thus, the nanoscale morphology of the thin films is crucial for both the geminate and non-geminate recombination within the BHJ composite active layer. One approach that is used to control the nanomorphology of blends is to introduce a solvent processing additive^15^,^16^,^17^, and this represents one of the simplest but the most effective methods.

However the fundamental operating mechanism and variation in the morphology of these all-polymer blends treated with binary solvent additives have yet to be thoroughly investigated. Furthermore, a kinetic description of the development of the P(NDI2OD-T2)-based BHJ morphology, particularly in solvent processing binary additive systems, has yet to be clearly presented. The crystalline behavior and miscibility of BHJ blends based on P(NDI2OD-T2) may be fundamentally different from those of their fullerene counterparts. Therefore, it is essential to define the parameters that govern the morphlogical development of P(NDI2OD-T2)-based solar cells.

To address this issue, we herein report on a PCE of 4.39% from all-polymer solar cells fabricated using a poly{[4,8-bis-(2-ethyl-hexyl-thiophene-5-yl)-benzo[1,2-*b*:4,5-*b′*]dithiophene-2,6-diyl]-*alt*-[2-(2-ethyl-hexanoyl)-thieno[3,4-*b′*]thiophen-4,6-diyl]}(PBDTTT-CT) and P(NDI2OD-T2) BHJ composite processed using the binary solvent processing additivies 1,8-diiodooctane (DIO) and 1-chloronaphthalene (CN). The quantum efficiency, film morphology, charge transport, and exciton diffusion were systematically studied to understand the factors that improve the final performance of the PBDTTT-CT:P(NDI2OD-T2) BHJ polymer solar cell devices. To the best of our knowledge, this work presents the one of the successful case where a binary processing additives was applied to fabricate all-polymer solar cell devices.

## Results and Discussion

**Figure 1:**
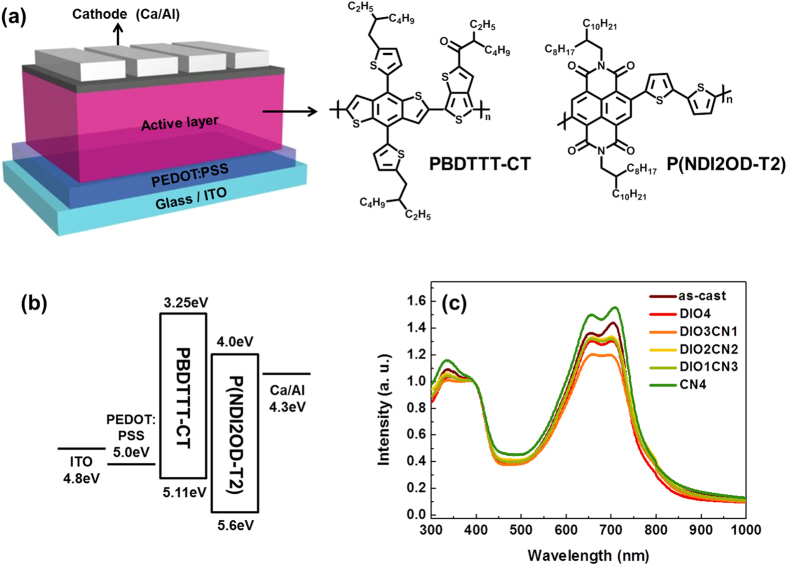
(**a**) Device architecture of the all-polymer BHJ solar cells; (**b**) the energy levels of the different BHJ components; (**c**) normalized absorption spectra of the PBDTTT-CT:P(NDI2OD-T2) blend film at various binary additive vol. ratios.

Figure 1a shows a schematic diagram of the present PBDTTT-CT:P(NDI2OD-T2) all-polymer devices, which employ a conventional architecture consisting of ITO/PEDOT:PSS/all-polymer BHJ active layer/Ca/Al. We applied PBDTTT-CT as the donor compound because it has been described as one of the most successful donor materials and it produces organic solar cells with an excellent performance^18^,^19^. Therefore, blending PBDTTT-CT + P(NDI2OD-T2) appears to be promising, a priori, to fabricate all-polymer solar cells.

In an energy level diagram, the highest occupied molecular orbital (HOMO) and the lowest unoccupied molecular orbital (LUMO) energy levels for PBDTTT-CT^20^ are more than 0.5 eV higher than those of P(NDI2OD-T2)^21^ (see Fig. 1b), indicating that the energy level positions of the donor and the acceptor are suitable for an efficient charge transfer and separation at the interface between these two polymers^22^.

At first, we report on the optical properties of PBDTTT-CT:P(NDI2OD-T2) blend films in order to distinguish the effects arising from the binary additive system. Figure 1c shows the UV-vis absorption spectra of the PBDTTT-CT:P(NDI2OD-T2) thin films at different DIO/CN vol. ratios. Clearly, the absorption spectra of the PBDTTT-CT and its blend with P(NDI2OD-T2) span the solar spectrum from visible light to 900 nm, which is sufficient to generate charges at the heterojunction^23^. Furthermore, the absorption spectra of the blend films provide information regarding the molecular ordering of the films. When PBDTTT-CT is blended with P(NDI2OD-T2) without additives, the absorption peaks are quite distinctly positioned in the ranges from 300 to 450 and from 600 to 750 nm. However, the difference becomes more pronounced when binary processing is performed with additives. It is quite clear that the P(NDI2OD-T2) absorption peaks (at ca. 715 nm) shift slightly toward blue at heavier CN loading up to 1 vol% (DIO 4 vol% and DIO/CN 3:1 vol%), and the originally strong vibronic peaks diminish significantly. In particular, the blend film processed with 3:1 DIO/CN binary additives showed the largest change, for which the film absorption peaks are considerably weakened. Such a decrease in the peak intensity and in the shape of the broader peaks indicates that the molecular interchains are interacting less weakly and that there is a reduced local structural order^24^. The loss of ordering was ascribed to the fact that 3:1 DIO/CN is finely dispersed on a molecular basis between the two polymer chains, thus preventing the compound from crystallizing^25^. It is therefore conjectured that the remarkable ability of the binary additives is related to its capability to redistribute PBDTTT-CT and P(NDI2OD-T2) in the composite film.

### Optimization of the performance of the photovoltaic devices by binary additives

**Figure 2:**
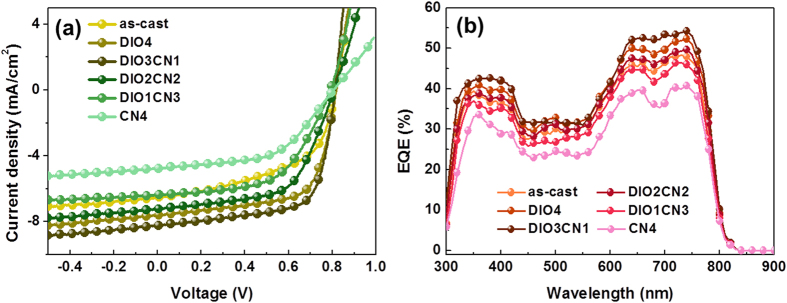
Effect of the binary additive in the *o*-DCB solution on the device properties of the PBDTTT-CT:P(NDI2OD-T2) solar cell: (a) current-voltage characteristics under simulated sunlight; (b) EQE spectra.

We carried out an optimization for the photovoltaic devices based on the PBDTTT-CT: P(NDI2OD-T2) with single additives and binary additives of different vol. ratios. [The optimized blend ratio of PBDTTT-CT to P(NDI2OD-T2) was 1:1, and the active layer thickness was of 81–89 nm.] The *J-V* characteristics of these devices processed from *o*-dichlorobenzene solvent are shown in Fig. 2a, and the performance parameters are listed in Table 1. In general, the co-additives of the films improved the performance, which is consistent with the results of numerous studies concerning the binary additive treatment of polymeric/small molecule or all-polymer systems. BHJ solar cells processed with binary processing additives exhibited a relatively stable open-circuit voltage (*V*oc) when a small amount of additive was added^26^,^27^, and in a similar manner, the *V*oc in our device, which is in the range from 0.80–0.82 V, was almost independent of the additive treatment. However, the behavior of the short-circuit current (*J*_SC_) and fill factor (FF) was more interesting since these values varied significantly. For the devices without additive treatment (as-cast), the *J*_SC_ and FF were only 6.53 mA cm^−2^ and 52.5%, respectively. After adding 4 vol% of DIO additive, the *J*_SC_ increased to 7.62 mA cm^−2^, and the FF increased to 63.6%.

Upon adding 3:1 DIO/CN to the solvent, the *J*_SC_ and the FF of the resulting blend film further increased to 8.20 mA cm^−2^ and 66.2%, which are among the best values reported for BHJ organic solar cells with a binary additive system. However, the *J*_SC_ started to drop when the CN concentration in the DIO/CN binary processing additives was higher than 1%, and the FF of these devices also behaved similarly in terms of the *J*_SC_. At a low CN concentration, the *J*_SC_ and the FF were quite high at around 8 mA cm^−2^ and 65%, respectively. When the CN concentration was higher than 1%, the *J*_SC_ and FF decreased to ≈6 mA cm^−2^ and ≈55%, respectively. The combined effect led to a best PCE of 4.39% for the 3:1 DIO/CN binary additives. Thus, as is argued below, we believe that the significant increase in the photovoltaic performance is due to the differences in the network morphology and the molecular packing that result from the optimized binary processing additive system.

Figure 2b shows the external quantum efficiency (EQE) data for the correction of the photocurrent. The EQE results are in good agreement with the *J*_SC_ values that are mentioned above. Evidently, the binary additives show a synergistic effect on the enhancement of the EQE. The EQE spectrum for the optimized film processed with the 3:1 DIO/CN additives displayed a peak EQE exceeding 59% at 720 nm and a broad photoresponse from 300–900 nm. In contrast, the EQE spectrum of the device prepared without or with a single additive exceeded 40% at 696 nm. Clearly, the enhanced EQE spectrum of the 3:1 DIO/CN binary additive cells could be attributed to a greater conversion of incident light into a photocurrent for all absorption wavelengths, which is consistent with the higher circuit current that was observed^28^.

### PBDTTT-CT:P(NDI2OD-T2) film morphology characterization

**Figure 3:**
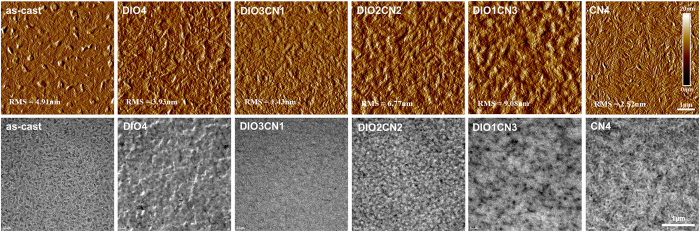
Upper panel: AFM phase images; lower panel: TEM images. In these panels, the results for the **PBDTTT-CT:P(NDI2OD-T2)** blend films as a function of binary additive ratios are presented.

The microstructure of the PBDTTT-CT:P(NDI2OD-T2) blends was investigated with a variety of characterization methods to understand the effects that the binary processing additives had in this novel all-polymer system. Atomic force microscopy (AFM) was employed to image the surface topography, and transmission electron microscopy (TEM) was used to image the bulk morphology. The phase images of the PBDTTT-CT:P(NDI2OD-T2) blends that were prepared using binary additives of different compositions are shown in Fig. 3 (upper panel), and the evolution of the morphology of the blended films can be thoroughly observed. As shown in Fig. 3, the thin film blends processed without additives formed sparse, coarse phase-separated domains with a root-mean-squared (RMS) roughness of 4.91 nm. These large coarse domains are considered to be non-ideal and thus unfavorable since a large extent of phase aggregation results in the limited availability of an interfacial area to allow for efficient exciton dissociation and carrier generation. Thus, these devices exhibit poor performance^29^. The fibril aggregation increased when 4 vol% DIO was used, as seen through the rough-coding of the aggregation, and although the distance of these aggregates decreased, the phase-separated network still remained. A dramatic change in the surface morphology was found for the PBDTTT-CT:P(NDI2OD-T2) film processed with 3:1 DIO/CN co-additives, with the film exhibiting a smooth and homogeneous surface morphology (surface roughness decreased to 1.43 nm). The nanoscale flat network and reduced phase separation became more apparent, implying that the two polymer matrixes form more sufficient and better-connected pathways^30^. This is also in agreement with the optical observations where both PBDTTT-CT and P(NDI2OD-T2) exhibited fewer crystallites in the film processed with 3:1 DIO/CN processing additives. As the CN content further increased to 2 and 3 vol% in the DIO/CN co-additives, the size of the aggregation, degree of the phase separation, and coarse features increased gradually relative to those for the films treated with the 3:1 DIO/CN additives. In addition, the RMS roughness also increased to 6.77 and 9.08 nm, respectively, which may imply an enhanced crystallinity for the two compounds in the heterojunction film. Wide angle X-ray scattering (GIWAXS) was carried out to inspect the macromolecular accumulation, and the results will be discussed below. In the 4 vol% CN film, the surface morphology exhibits characteristics that are unlike those of other blend films, such as a liquid crystal-like morphology. We believe that this “slash”-like pattern might be due to compound crystallites.

Likewise, the TEM images of the PBDTTT-CT:P(NDI2OD-T2) blend films treated with different DIO/CN vol. ratios revealed a similar behavior as in the phase AFM images. Obviously, the as-cast film exhibits a fibrillary packing morphology with a finer scale and elongated phases. Nevertheless, the device with this pristine film exhibited poor device performance because an over-aggregation of the phases had not been observed in the high-magnification TEM images. With respect to the 3:1 DIO/CN devices, the TEM image clearly shows a considerably homogeneous bulk morphology without distinct aggregated domains. The results therefore indicate that the 3:1 DIO/CN co-additives can help one polymer penetrate into the other polymer matrix, resulting in better intermixing and finer phase-separated domains. With the decrease in the DIO content (2:2 DIO/CN and 1:3 DIO/CN binary additives), the fraction of the TEM images occupied by the darker phase increases, indicating that this darker phase is rich in PBDTTT-CT^31^.

In other words, the AFM images and the TEM results are correlated with the performance of the solar cell devices. With an improved percolating phase, the electron-hole pair would be easily dissociated since a greater interface area between the donor and acceptor molecules can be obtained. In addition, the reduction in the domain size and lower extent of the phase separation would allow excitons to more easily diffuse to the donor/acceptor interfaces and to subsequently generate free carriers. This would also result in fine interpenetrating networks in the entire bulk heterojunction forming pathways for efficient charge transport and collection. Hence, charge extraction and injection are significantly improved, as is strongly indicated by the improvement in *J*_SC_ and FF in the PBDTTT-CT:P(NDI2OD-T2) device processed with the 3:1 DIO/CN binary additives^32^,^33^.

### 2D-GIWAXS study on the nanostructural order of pristine polymer and polymer/polymer blend thin films

**Figure 4:**
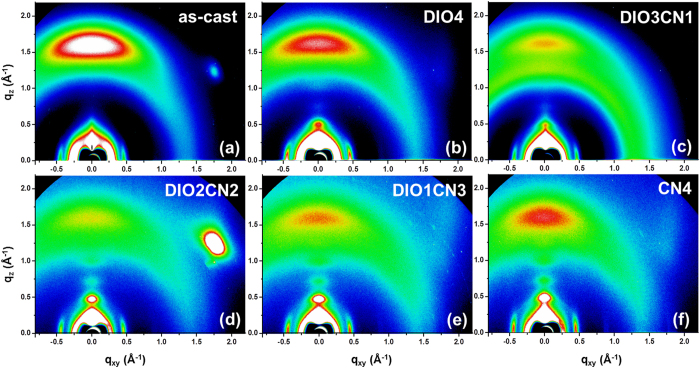
Two-dimensional GIWAXS images of the PBDTTT-CT:P(NDI2OD-T2) films with different additive treatments: (a) no additive, (b) DIO 4, (c) DIO/CN 3:1, (d) DIO/CN 2:2, (e) DIO/CN 1:3, and (f) CN 4.

Although the AFM and TEM images reveal dramatic morphological features, it is difficult to gain insight into the degree of crystallinity from the AFM and TEM measurements alone. Therefore, we also conducted two-dimensional grazing incidence wide-angle X-ray scattering (2D-GIWAXS) on the same films that had been used for the AFM characterization (Fig. 4). To accurately investigate the peak position and the intensity of all of the blend films in the GIWAXS patterns, we configured these images to have the same color densities (same brightness and contrast).

The pristine PBDTTT-CT:P(NDI2OD-T2) film shows a broad (0 0 1) reflection peak at q_xy,z_ ≈ 0.26 Å^−1^ and a lateral scattering signal at q_xy_ ≈ 0.47 Å^−1^. Also, a distinct signal is present at q_z_ ≈ 1.62 Å^−1^ in the out-of-plane direction, and it is indicative of some face-on π-π stacking^34^. Interestingly, the increase in the CN vol. ratios resulted in two scattering reflections at q_xy_ ≈ 0.47 Å^−1^ and q_z_ ≈ 1.62 Å^−1^ to remain at the same positions but with intensities that changed according to the same trends in that the intensities of the two signals gradually decrease for the 3:1 DIO/CN co-additive films relative to those of the as-cast samples, but the intensities of these reflections continuously increase upon further adding CN additive from 2 vol% to 4 vol% again. In the case of the 4 vol% DIO treated with PBDTTT-CT:P(NDI2OD-T2) blends, we are able to observe a (0 0 2) (q_z_ ≈ 0.52 Å^−1^) peak, as shown in Fig. 4b. In the 3:1 DIO/CN binary additive films, this (0 0 2) diffraction peak was even weakened. Based on the results above, we can conclude that the polymer chains in the 3:1 DIO/CN additive treatment formed a ‘dominantly amorphous structure’ in the blend films. Consequently, processing the blends with 3:1 DIO/CN co-additives decreases the solid state order of both the donor and the acceptor, perhaps in part explaining the increase in the intermixing of the two compounds and in the *J*_SC_ of these blends^35^. When more than 2 vol% CN was used, the GIWAXS spectrum shows different reflections arising from main-chain crystallization. In particular, distinct (0 0 *h*) diffraction peaks are sharply seen along the q_z_ direction, indicating that a crystalline structure arises in the edge-on orientation^36^. Moreover, with the increase, the (0 0 *h*) Bragg peaks became clearer and stronger. These results imply that more than 2 vol% CN treatment improves the crystalline stacking structure, and these observations are completely consistent with the results shown in Fig. 3.

**Figure 5:**
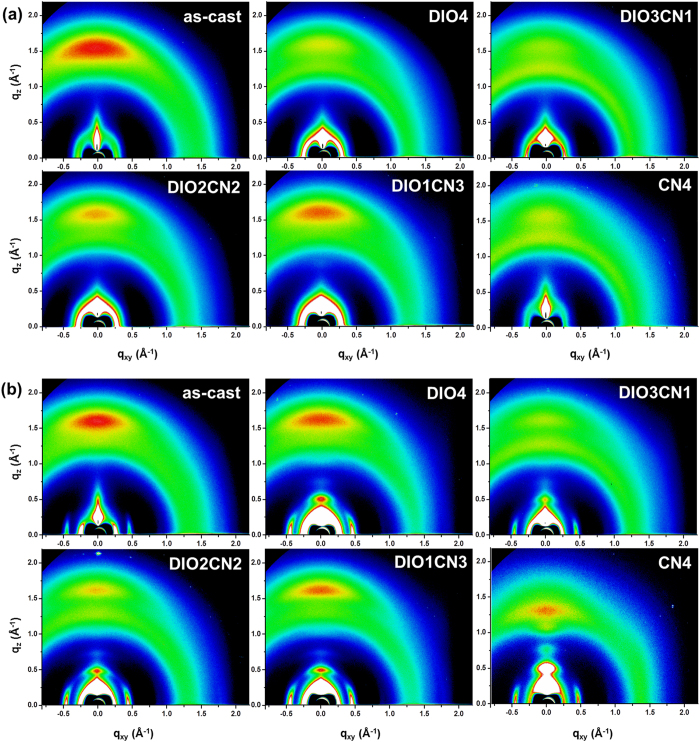
2D-GIWAXS patterns for pure PBDTTT-CT (upper line) and P(NDI2OD-T2) (lower line) films from different solutions.

In order to understand the origin of the binary processing additives, we further focus our attention on the crystalline texture of the net polymer films made with PBDTTT-CT and P(NDI2OD-T2). The pure polymer therefore serves as an ideal model polymer to study mixing with co-additives since any changes concerning the crystallinity of two polymers or within the interlamellar domains can be tracked. Therefore, the effect of the binary processing additives on the crystallization of each polymer was also studied using 2D-GIWAXS (Fig. 5). Figure 5a shows that there is a strong preference diffraction for face-on packing of crystallites (q_z_ ≈ 1.53 Å^−1^, π-π stacking spacing = 4.10 Å) and a polymorph (0 0 1) peak (q_xy,z_ ≈ 0.26 Å^−1^ in the as-cast blend. Upon adding 4% DIO, the PBDTTT-CT polymorph peak appears in the more lateral direction, and a clear π-π stacking peak disappears, indicating that lamellar structures formed in the polymer with a more random orientation with a reduction in the solid state face-on order^37^. In the 3:1 DIO/CN processed PBDTTT-CT films, much fewer crystalline reflections appear relative to the GIWAXS spectrum presented from the 4 vol% DIO additive treatment. This implies that the 3:1 DIO/CN co-additives can also disturb the crystalline packing structure of the pure PBDTTT-CT polymer. When the CN concentration was higher than 1%, the PBDTTT-CT crystallites increased, suggesting that the interlayer interaction became stronger.

The trend of the GIWAXS spectrum resembles the scattering patterns of pure PBDTTT-CT films, however, the variation in the GIWAXS patterns is greater in P(NDI2OD-T2) as a function of the binary additive ratios (Fig. 5b). P(NDI2OD-T2) films processed with 4 vol% DIO shows a quite different reflection compared to pristine P(NDI2OD-T2) film with a distinct scattering signal at q_z_ ≈ 0.52 Å^−1^ [a (0 0 2) reflection peak] along the out-of plane direction. A strong lateral diffraction peak appears at q_xy_ ≈ 0.47 Å^−1^ with a broad polymorph peak (q_xy,z_ ≈ 0.26 Å^−1^). The intensity of these all-scattering peaks significantly diminishes for the 3:1 DIO/CN processing additives, implying fewer molecular packing^37^. This result is quite similar to that found with neat PBDTTT-CT films. The reflection peaks remain at the same positions, but their intensities considerably increase with the addition of the binary additives, beginning at higher than 2 vol% CN, which indicates an increase in the oriented P(NDI2OD-T2) crystalline domains. Surprisingly, upon adding a single CN 4% additive, strong (0 0 *h*) diffraction peaks can be seen in the out-of-plane direction with a Bragg distance of a corresponding q value of 24.17 Å^−1^. These signals demonstrate that much stronger P(NDI2OD-T2) crystallites swamped the BHJ blends with the edge-on orientation.

Qualitatively, the GIWAXS patterns of all PBDTTT-CT:P(NDI2OD-T2) BHJ prepared with binary processing additives resemble the scattering patterns of the individual components, indicating that the polymers crystalize in a similar manner. The evidence shown further indicates that the polymer lamella correlation diffraction for all PBDTTT-CT:P(NDI2OD-T2) can be seen to follow the same situation as that in neat polymer films with a π-π stacking scattering peak around q_z_ ≈ 1.53 Å^−1^ arising from both PBDTTT-CT and P(NDI2OD-T2), which proved to be too close together and overlapping in the blend films. A Bragg diffraction (0 0 *h*) signal from the P(NDI2OD-T2) neat films strongly appears with a crystalline structural order for the edge-on orientation. Moreover, the lateral reflection (q_xy_ ≈ 0.47 Å^−1^) in the in-plane direction also arises from pure P(NDI2OD-T2). Therefore, the GIWAXS images of all of the BHJ blends exhibit a stronger variation in the P(NDI2OD-T2) crystallites by the co-additives, and thus we can conclude that binary processing additives have a greater influence on the P(NDI2OD-T2) crystallinity in the PBDTTT-CT:P(NDI2OD-T2) blends.

### Photophysics for exction diffusion and charge separation

The photoluminescence (PL) of photoactive donors can be efficiently quenched when intimately blended with an acceptor due to the photoinduced charge transfer, and the geminate recombination of the initially generated charge transfer states back to the ground state may compete with the dissociation of these charges into free charge carriers^38^. PL quenching is a simple method that can be used to investigate whether the excitons in both materials successfully reach the interface. Also, a blend with a smaller domain size has a higher PL quenching efficiency compared to that of a big one. Thus, the PL quenching efficiency can directly reflect the active domain size in a blend^39^.

**Figure 6:**
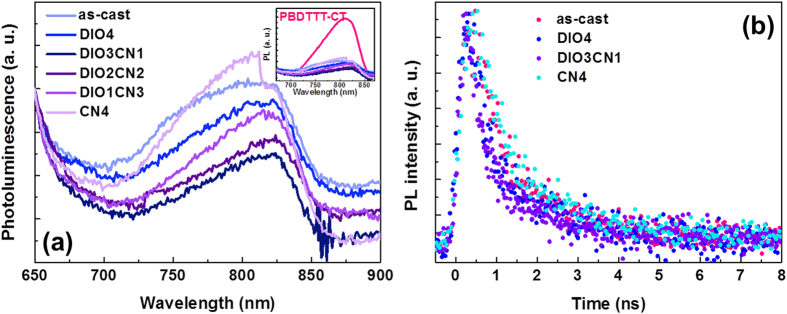
Effect of the binary processing additives on the PBDTTT-CT:P(NDI2OD-T2) blend films. Steady-state photoluminescence spectra (excitation was at 630 nm) (**a**) and photoluminescence decay profile (**b**).

Here, the PL spectra of the PBDTTT-CT:P(NDI2OD-T2) blends with a treatment of different DIO/CN vol. ratios of 0:0, 4:0, 3:1, 2:2, 1:3, and 0:4 were measured and are shown in Fig. 6a. Interestingly, the PBDTTT-CT:P(NDI2OD-T2) film with 3:1 DIO/CN co-additives showed the lowest level of photoluminescence among all samples as a result of the significant exciton quenching at the PBDTTT-CT/P(NDI2OD-T2) interface (The PL quenching efficiency exhibited the highest value of ca. 93%, see an insert figure of Fig. 6a). These results also indicate that the 3:1 binary additive films have a sufficiently optimal morphology for exciton dissociation and, thus, a high *J*_SC_, which is consistent with results observed for the morphology. Furthermore, it is evident that in the PBDTTT-CT:P(NDI2OD-T2) blend films with the 3:1 DIO/CN additive treatment, most excitons generated in the PBDTTT-CT were able to diffuse to the donor:acceptor interface.

To learn more of the charge separation in the PBDTTT-CT:P(NDI2OD-T2) blend films, we performed a time-correlated single-photon counting measurement, as shown in Fig. 6b. The PL decay profiles of the PBDTTT-CT:P(NDI2OD-T2) films were fitted well with bi-exponential decay fitting^40^, which suggests that the PL exciton decay in PBDTTT-CT occurred through two relaxation pathways. Table 2 summarizes the PL lifetime results of the PBDTTT-CT:P(NDI2OD-T2) film as a function of the binary additive ratios. In Fig. 6b, the time-resolved PL of all blend films under different conditions are quenched by the P(NDI2OD-T2), which is consistent with the static PL spectra shown in Fig. 6a. The decay values indicate that the average photoluminescence decay time (exciton lifetime) can be calculated to be of 2.75 ns for the as-cast films, 1.70 ns for the 4 vol% DIO films, 1.46 ns for 3 vol% DIO:1 vol% CN films, and 2.82 ns for 4 vol% CN films. The shorter PL lifetime of a donor-acceptor combined cell implies that faster carrier dynamics in the BHJ cells are a result of the improvement in the efficiency of the cell. Thus, the exciton quenching efficiency of the PBDTTT-CT:P(NDI2OD-T2) films increase due to processing with 3:1 DIO/CN co-additives, which is consistent with the results of the 2D-GIWAXS measurements and the BHJ solar cell tests.

### SCLC studies on the carrier mobility of the blend thin films

**Figure 7:**
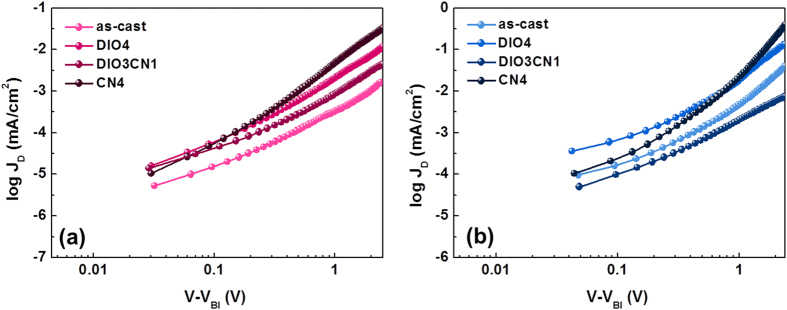
*J-V* characteristics measured according to the space charge limited current (SCLC) method with PBDTTT-CT:P(NDI2OD-T2) films in the dark. (**a**) hole-only devices, (**b**) electron-only devices.

The charge carrier mobility in the blend films is critical in the BHJ solar cells because the photogenerated charges that are extracted at the electrode depend on the competition between the carrier sweep out, which is limited by the carrier mobility, and the loss of photogenerated carriers due to recombination^41^. The charge transport properties of the PBDTTT-CT:P(NDI2OD-T2) blends are assessed by fabricating hole-only and electron-only diodes, measuring the dark current density-voltage (*J-V*) characteristics, and then analyzing the results using a space-charge-limited current (SCLC) model. Figure 7 shows the *J-V* curves of the hole-only device in the configuration of the ITO/PEDOT:PSS/blend/Au (Fig. 7a) and the electron-only device in the configuration of the Al/blend/Al (Fig. 7b) (The carrier mobility data are reported in Table 3).

The experimental results were fitted, revealing that the pristine PBDTTT-CT:P(NDI2OD-T2) blend had hole and electron mobilities of 5.12 × 10^−6^ cm^2^ V^−1^ s^−1^ and 7.64 × 10^−5^ cm^2^ V^−1^ s^−1^, respectively. When a single 4 vol% DIO or CN additive was introduced, the hole mobility increased to 7.40 × 10^−6^ cm^2^ V^−1^ s^−1^ and 8.64 × 10^−6^ cm^2^ V^−1^ s^−1^, respectively. The increase in hole mobility with DIO or CN content is in good agreement with the results obtained through the nanoscale morphology analysis, in which the domain size and the crystallinity were observed to increase when a single additive was introduced into the solvent^42^. Similarly, the electron mobility also increased to 9.11 × 10^−5^ cm^2^ V^−1^ s^−1^ for the PBDTTT-CT:P(NDI2OD-T2) films processed from 4 vol% DIO and 7.32 × 10^−4^ cm^2^ V^−1^ s^−1^ for the 4 vol% CN treatment, which is consistent with the increase in the P(NDI2OD-T2) solid state order observed through the GIWAXS measurements. Surprisingly, the lowest hole and electron mobilities were observed in the PBDTTT-CT:P(NDI2OD-T2) films with 3:1 DIO/CN binary processing additives. We speculate that the binary additives facilitate the percolating network of the polymer domains and, hence, the charge carrier mobility relatively decreases when the 3:1 content DIO/CN is used. In addition, the electron and hole mobilities are more balanced in the blend film processed with 3:1 DIO/CN binary processing additives. The balanced carrier transports perhaps contribute to the higher device performance because the accumulated SCLC charges, and hence the recombination processes, are reduced by the increase in carrier mobility and improvement in charge collection efficiency^43^,^44^.

### Effects of recombination on efficiency

**Figure 8:**
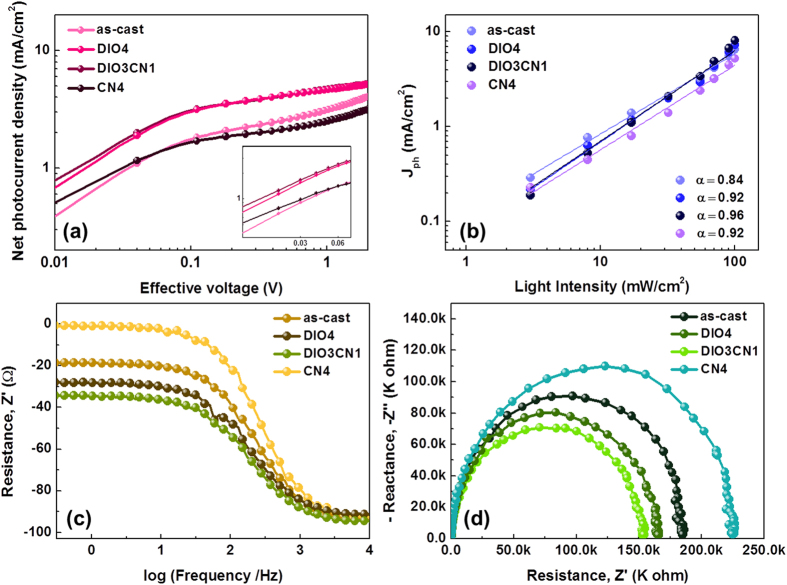
(**a**) Photocurrent density versus effective voltage (J_ph_ − V_eff_) characteristics of a PBDTTT-CT:P(NDI2OD-T2) solar cell that process with various binary additives. (**b**) The measured *J*_SC_ plotted against the light intensity (symbol) on the logarithmic scale and the fitted power law (line) yield α. The equivalent circuit model of the (**c**) reactance-frequency, and (**d**) the Cole-Cole plots of the devices in different binary additive conditions.

The binary additive system in the active blend network also improved the charge collection under working conditions. Figure 8a shows the *J-V* characteristics for a wide reverse bias range under an AM 1.5G illumination. The results are plotted using the net photocurrent, *J*_ph_ = *J*_L_ – *J*_D_ (where *J*_L_ and *J*_D_ are the current density under illumination and in the dark, respectively), and the dependence on the effective applied voltage, V_eff_ = V_0_ – V (where V is the applied voltage, and V_0_ is the voltage at which *J*_ph_ = 0)^45^. The influence of the charge extraction dominates at low voltages while the influence of the charge generation dominates at high voltages^46^. For a large reverse voltage (V_eff_ > 1.2 V), *J*_ph_ becomes saturated, with a flat plateau for the 4 vol% DIO and 3:1 vol% DIO/CN treated devices, which overlap each other. However, those of the PBDTTT-CT:P(NDI2OD-T2) cells with 4 vol% CN additive and without additive treatment increased gradually. The difference in the saturation region shows that greater charges are generated in the devices processed with 4 DIO and 3:1 DIO/CN additives.

Interestingly, for a low effective voltage range (V_eff_ < 0.1 V), the *J*_ph_ − V_eff_ characteristics for the two devices (4 vol% DIO and 3:1 vol% DIO/CN treatment) have large differences. Since the *J*_ph_/*J*_sat_ ratio is essentially the product of the exciton dissociation efficiency and the charge collection efficiency, a decrease in *J*_ph_/*J*_sat_ suggests either a reduction in the exciton dissociation efficiency or a decrease in the charge collection efficiency^47^. A reduction in the charge collection efficiency suggests that bimolecular recombination begins to dominate, and this usually leads to a lower FF^48^. A higher *J*_ph_ − V_eff_ characteristics from the 3:1 DIO/CN processed device identifies the effect of the optimal DIO/CN binary additive ratios on the reduction of the bimolecular recombination and more efficiently sweeping out free charges at a low effective voltage, at which the maximum power output condition of the solar cells occurs.

We studied the variation in the photocurrent (*J*_ph_) as a function of the illumination intensity in order to gain deeper insight into the charge recombination kinetics. The light intensity-dependent *J*_ph_ were measured under various light intensities from 100 to 3 mW/cm^2^, and the relative *J*_ph_ at V = 0V are plotted against the light intensity in Fig. 8b. The relationship between *J*_ph_ and the intensity of the light can be represented using the power law equation (equation (1)):


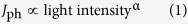


where α is indicative of efficient sweep-out of carriers prior to recombination^49^. At short circuit, the bimolecular recombination should be minimum (α~1) for a maximum carrier sweep out, and any deviation from α~1 implies nongeminate recombination^49^.

From Fig. 8b, we determined the exponential factor (α) for the device processed with 0%, 4% DIO, 3% DIO:1% CN, and 4% CN additive to be 0.84, 0.92, 0.96 and 0.92, respectively. A value closer to unity (α = 0.96) in the device processed with 3:1 binary additive treatment implies that the sweep-out is the most effective in the device. In other words, all charge carriers in this system have been more separated and transported prior to recombination, which is well correlated with a balanced charge transport observed from the mobility study. The balanced hole and electron mobility in these devices, as discussed earlier, can also be attributed to the proximity of the α value to unity.

Impedance measurements were performed in order to extract further information related to the defect states and their role as trapping and/or recombination centers. Defect states might have detrimental effects on the performance of the solar cells. Charges defects alter the profile of the electrical field and may reduce the drift driving force for carrier transport, consequently diminishing carrier collection at the contacts^50^,^51^. The measurements were performed following known techniques to characterize organic solar cells.

Figure 8c shows the resistance vs. frequency spectra for *V*oc in the dark. The resistance for each device decreases as the frequency increases from 100 Hz to 1 MHz. These results imply that there should be at least one capacitance shunted with the close to short circuit condition while the frequency increases^52^. Furthermore, the Cole-Cole plot in Fig. 8d is not really a smooth semicircle, which indicates that there are more than one resistance-capacitance-shunt pairs in the circuit system. The internal series resistance of the solar cells is composed of the sheet resistance of the electrodes and the charge transport resistance at the BHJ/electrode interfaces or inside the BHJ active layer. Since all-polymer solar cells have the same device structure, only the BHJ composite layer is processed under different conditions. Thus, the sheet resistance inside all these cells can be assumed to be the same, but the series resistance can provide us information related to the charge transport resistance that is affected by the solvent additives.

As expected, the similar shape and size of the region of the semicircles with the lower resistance for all devices implied that there were no differences in the interlayers or in the interfaces between the BHJ and the interlayer. However, we observe that the semicircle in the PBDTTT-CT:P(NDI2OD-T2) device increases with or without a single processing additive, indicating an increase in the capacitance and resistance in the active layer. This observation is consistent with the increase in the trap states, as was stated above^53^. The lowest internal series resistance is observed for the device processed with 3:1 DIO/CN additives, which suggests that the thin film morphology of the BHJ composite greatly improved due to the reduction in the phase separation and improvement in the percolating network due to the assistance of the binary solvent processing additives. These results are consistent with the previous observations of Figures 3 and 4.

## Conclusions

In conclusion, two functional additives were incorporated into the processing solvent, we were able to create efficient BHJ all-polymer solar cells with PBDTTT-CT:P(NDI2OD-T2) as an active layer. The approach described here provides an operationally simple and versatile tool that is available to tailor the heterojunction of the solar cell morphology in the systems. Interestingly, we observed a sharp elevation in the performance of the solar cells and the corresponding changes upon processing with the 3:1 DIO/CN binary processing additive (3 vol% DIO, 1 vol% CN and 96 vol% *o*-DCB) were observed through different characterization techniques. The PCEs significantly increased from 2.81% to 4.39%.

The microstructure and the photophysics of this system were examined in depth, which revealed that the binary additive treatments reduced the aggregation of the polymer compound, improved donor/acceptor mixing, achieved an efficient charge transfer, and led to balanced charge transport. Furthermore, the recombination loss mechanisms were proven to have been drastically reduced by decreasing the nongeminate recombination to a low level.

First, to understand the origin of the binary processing additives, we focus our attention on performing a GIWAXS analysis of neat PBDTTT-CT and P(NDI2OD-T2) with respect to the composition of the co-additives. Although more work is required, this result might shed light on one of the mechanisms that increases the performance of polymer-polymer blends. Future spectroscopic studies of photovoltaic blends with well-defined morphologies should be able to confirm these results. Therefore, our work provides a new way to distinctly characterize and regulate the morphologies of the PBDTTT-CT:P(NDI2OD-T2) blend structures to produce highly-efficient all-polymer solar cells.

## Experimental Section

### Materials

PBDTTT-CT and P(NDI2OD-T2) were obtained from 1-Material, Inc. and Polyera Corp., respectively, and were used as received. The molecular weight was *M*_w_ = 64000 g mol^−1^ for PBDTTT-CT and *M*_w_ = 174000 g mol^−1^ for P(NDI2OD-T2). All of the solvents where purchased from Sigma Aldrich, except for 1-chloronaphthalene from Alfa Aesar.

### Photovoltaic Device Fabrication and Characterization

The photovoltaic devices were fabricated with an ITO/PEDOT:PSS/PBDTTT-CT:P(NDI2OD-T2)/Ca/Al geometry through the following fabrication steps. Patterned ITO substrates were sequentially cleaned in an ultrasonic bath consisting of detergent, de-ionized water, acetone, and isopropanol. The cleaned ITO substrates were then subjected to UV/ozone treatment at room temperature for 30 min. A thin layer (~40 nm) of PEDOT:PSS (Clevios P VP AI 4083, filtered at 0.45 μm PVDF) was spin-coated onto the ITO surface at 4000 rpm. After baking at 120 °C for 20 min, the substrates were transferred into a nitrogen-filled glovebox. The PBDTTT-CT:P(NDI2OD-T2) (PBDTTT-CT:P(NDI2OD-T2) = 1:1 by weight) BHJ composite was spin-coated onto the PEDOT:PSS layer from (a) pristine *o*-DCB (*o*-DCB = 100 vol%); (b) *o*-DCB mixed with 4% DIO solution (*o*-DCB = 96 vol% and DIO = 4 vol%); (c) *o*-DCB mixed with 3% DIO and 1% CN solution (*o*-DCB = 96 vol%, DIO = 3 vol% and CN = 1 vol%); (d) *o*-DCB mixed with 2% DIO and 2% CN solution (*o*-DCB = 96 vol%, DIO = 2 vol% and CN = 2 vol%) ; (e) *o*-DCB mixed with 1% DIO and 3% CN solution (*o*-DCB = 96 vol%, DIO = 1 vol% and CN = 3 vol%; (f) *o*-DCB mixed with 4% CN solution (*o*-DCB = 96 vol% and CN = 4 vol%). Then, a negative Ca (~10 nm)/ Al (~100 nm) electrode was thermally evaporated on the active layer under a shadow mask at a base pressure of about 2 × 10^–6^ Torr. The active layer of the solar cells had a size of 0.09 cm^2^. The current density-voltage (*J-V*) characteristics of the photovoltaic devices were measured under ambient conditions using a Keithley Model 2400 source measurement unit. An Oriel xenon lamp (450 W) with an AM 1.5G filter served as the solar simulator, and the light intensity was calibrated to 100 mW/cm^2^ using a silicon cell with a KG5 filter calibrated by the National Renewable Energy Laboratory (LREL). The solar cells were tested under various light intensities by modulating the intensity of the light with a series of two neutral density filter wheels with six filters each, allowing for up to 15 steps of intensity from 100 to 5 mW/cm^2^. The intensity of the light transmitted through the filter was independently measured by using a power meter. The EQE spectra were obtained from a photomodulation spectroscopic setup (model Merlin, Oriel), a calibrated Si UV detector, and an SR570 low-noise current amplifier.

### Optical Absorption and Photoluminescence Quenching Measurements

All absorption measurements were performed using a Cary 5000 UV-Vis-NIR double-beam spectrophotometer in the two-beam transmission mode. The background absorption was subtracted by illuminating the sample with one beam and a reference with the other beam. The PL spectra were measured for the pure PBDTTT-CT and PBDTTT-CT:P(NDI2OD-T2) films processed with binary additives spin-coated onto quartz substrates by using a calibrated fluorescence spectrophotometer (FP-6500, JASCO). The photoluminescence of these all-polymer blend films upon excitation at 630 nm.

### Time-Correlated Single Photon Counting (TCSPC)

The PL life time profiles of the PBDTTT-CT:P(NDI2OD-T2) films were obtained by using the time-correlated single-photon counting method. The output of a home-built cavity-dumped Kerr lens mode-locked Ti:sapphire laser running at 820 nm was doubled to generate excitation pulses at 410 nm, and the fluorescence at the magic angle was detected by using a thermoelectrically cooled microchannel plate photomultiplier tube (Hamamatsu, R3809U-51). The instrument response function had a full width at half maximum of 42 ps to provide ~8 ps time resolution with deconvolution. A sample cuvette was attached to a home-made moving stage in order to minimize the photodamage. All experiments were carried out at room temperature.

### AFM, TEM and GIWAXS measurements

The AFM (Multimode IIIa, Digital Instruments) was operated in the tapping mode to obtain surface images (surface area: 5 × 5 μm^2^) of the blend films under ambient conditions. The TEM images were obtained using a HITACHI-7600 operated at 100 kV. The active layers were floated from the water-soluble PEDOT:PSS substrate onto the surface of dematerialized water and picked up with 200-mesh copper TEM grids.

For the structural analysis, two-dimensional GIWAXS specular scans were obtained by using the 3C beam lines at the Pohang Accelerator Laboratory. The 2D-GIWAXS measurements were carried out with a sample-to-detector distance of 220.761 mm. The data were typically collected for ten seconds by using an X-ray radiation source at λ = 1.1189 nm with a 2D charge-coupled detector (CCD) (Roper Scientific, Trenton, NJ, USA). The samples were mounted on a home-built z-axis goniometer equipped with a vacuum chamber. The incidence angle a_i_ of the X-ray beam was set at 0.13°, which is an intermediate value between the critical angles of the films and the substrate (a_c,f_ and a_c,s_). The samples were prepared for the X-ray measurements by spin-coating pure polymer and polymer blend films onto PEDOT:PSS spin-coated Si wafers.

### SCLC Measurements

Hole-only devices were fabricated through the following procedure. The PBDTTT-CT:P(NDI2OD-T2) layer was spin-coated on separate ITO substrates covered with 40-nm-thick PEDOT:PSS, which acted as the anode. A gold electrode (Au, 100 nm) was then vacuum-deposited on each layer as the cathode. The Au layer was deposited at a low speed (1 s^*–*1^) to avoid the Au atoms penetrating into the active layer. Electron-only devices with an Al/PBDTTT-CT:P(NDI2OD-T2)/Al architecture were also fabricated. The mobilities were extracted by using the Mott–Gurney relationship (SCLC) (equation. (2)) to fit the current–voltage curves in the range from 0 to 4 V


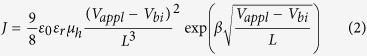


where *J* represents the current density, *L* represents the film thickness of the active layer, *μ*_h_ represents the hole mobility, *ε*_0_*ε*_r_ represents the dielectric permittivity of the active layer, *V*_appl_ is the applied voltage, *V*_r_ represents the voltage drop due to the contact resistance and the series resistance across the electrodes, *V*_bi_ is the built-in voltage, and *β* is the field activation factor.

### Electrical Impedance spectroscopy

The electrical impedance spectra were measured using an impedance analyzer (BioLogic, SP-300) with a frequency ranging from 100 Hz to 1 MHz, and were analyzed using the Z-view software. The all-polymer solar cells were held at their respective open circuit potentials, which were obtained from the *J-V* measurements, while the impedance spectroscopy spectra were recorded.

## Additional Information

**How to cite this article**: Kim, Y. J. *et al.* A Mechanistic Understanding of a Binary Additive System to Synergistically Boost Efficiency in All-Polymer Solar Cells. *Sci. Rep.* **5**, 18024; doi: 10.1038/srep18024 (2015).

## Figures and Tables

**Table 1:** Summary of the performance of the PBDTTT-CT:P(NDI2OD-T2) devices from *o*-DCB solution without additive treatment and with additive treatment.

Active layer	Condition	*V*oc (V)	*J*sc (mA /cm^2^)	FF (%)	PCE^max^ (%)	PCE^avg^ (%)
PBDTTT-CT : P(NDI2OD-T2)	as-cast	0.82	6.53	52.5	2.81	2.74 ± 0.1
	DIO4	0.81	7.62	63.6	3.92	3.82 ± 0.3
	DIO3CN1	0.81	8.20	66.2	4.39	4.16 ± 0.3
	DIO2CN2	0.80	7.29	59.8	3.48	3.37 ± 0.1
	DIO1CN3	0.81	6.22	56.7	2.85	2.68 ± 0.2
	CN4	0.80	4.80	53.3	2.04	1.90 ± 0.2

PCE^avg^: data obtained from 8 devices.

**Table 2:** PL lifetimes of the PBDTTT-CT:P(NDI2OD-T2) films processed with different binary additives^a^.

Blend films	Condition	τ_1_[ns](*f*_1_)	τ_2_[ns](*f*_2_)	τ_avg_[ns]^b^	χ^2^^c^
PBDTTT-CT : P(NDI2OD-T2)	as-cast	4.18 (0.61)	0.52 (0.39)	2.75	1.041
	DIO4	2.44 (0.66)	0.28 (0.33)	1.70	1.092
	DIO3CN1	2.18 (0.62)	0.28 (0.38)	1.46	0.931
	CN4	4.46 (0.59)	0.49 (0.40)	2.82	1.044

^a^Monitoring wavelength is of 820 nm. τ_1_ and τ_2_ are the lifetimes (ns), *f*_1_ and *f*_2_ are the fractional intensities.

^b^τ_avg_ is the average lifetime obtained from *f*_1_τ_1_ + *f*_2_τ_2_.

^c^χ^2^ is the reduced chi-square value.

**Table 3:** Charge carrier mobility measured by the SCLC model.

Active layer^a^	Condition	*μ*_h_ (cm^2^/V s)	*μ*_e_ (cm^2^/V s)	*μ*_e_/*μ*_h_
PBDTTT-CT : P(NDI2OD-T2)	as-cast	5.12 × 10^−6^	7.64 × 10^−5^	16.87
	DIO4	7.40 × 10^−6^	9.11 × 10^−5^	12.31
	DIO3CN1	6.53 × 10^−6^	1.22 × 10^−5^	1.86
	CN4	8.64 × 10^−6^	7.32 × 10^−4^	84.72

^a^The thickness of PBDTTT-CT:P(NDI2OD-T2) blend films was *ca.* 81–89 nm: as-cast = 86 ± 1 nm, DIO4 = 83 ± 1.2 nm, DIO3CN1 = 82 ± 0.7 nm and CN4 = 88 ± 1 nm.
